# An Ecological Analysis of Online Medical Consumption Discourse Among Visually Impaired Individuals Using a Theory-Driven LLM Approach

**DOI:** 10.3390/healthcare14091132

**Published:** 2026-04-23

**Authors:** Woo-Hyuk Kim, Eunhye Park

**Affiliations:** 1 Department of Consumer Science, College of Commerce and Public Affairs, Incheon National University, Incheon 22012, Republic of Korea; woohyuk@inu.ac.kr; 2 Department of Food and Nutrition, College of BioNano Technology, Gachon University, Seongnam-si 13120, Republic of Korea

**Keywords:** visually impaired individuals, social ecological model, medical consumption, online discourse

## Abstract

**Background:** This study examines how medical consumption is discussed in online communities among individuals who are blind or visually impaired using the Social Ecological Model (SEM) to capture multilevel healthcare experiences within digital discourse. **Methods:** A total of 428 posts and comments were collected from Reddit’s r/Blind community. Term frequency–inverse document frequency keyword extraction and a theory-driven LLM-based classification approach were applied to categorize texts into five SEM levels: intrapersonal, interpersonal, institutional, community, and public policy. **Results:** The findings show that intrapersonal (44.4%) and public policy (29.8%) levels were the most prominent, indicating a strong emphasis on personal coping experiences alongside structural constraints in healthcare access. Institutional-level discourse accounted for 15.8%, whereas interpersonal (6.2%) and community (3.8%) discourse were relatively limited. Keywords and qualitative analyses revealed themes related to emotional adaptation, social support, service accessibility, mobility constraints, and welfare policy barriers. The Jaccard similarity analysis indicated stronger associations between institutional and policy levels, whereas community-level discourse remained relatively distinct. **Conclusions:** These findings highlight the importance of understanding healthcare experiences, both individually and structurally, in online environments. This study also demonstrated the potential of integrating LLM-based classification with theory-driven frameworks to enable an interpretable and scalable analysis of complex health-related discourse.

## 1. Introduction

Improving health-related quality of life and ensuring equitable participation in healthcare and social systems for people with disabilities remain central priorities in public health and health education [1,2]. Despite advances in medical technology and the expansion of inclusive policies, individuals with disabilities continue to experience persistent disparities in healthcare access, institutional support, and social recognition across disability types [3,4]. These disparities are particularly consequential for health education, as they shape health-related behaviors, decision-making processes, and opportunities for sustained engagement with healthcare systems [5,6].

In this context, understanding how individuals engage with healthcare systems is necessary to improve accessibility and utilization of medical resources as well as elucidate how these resources are consumed in practice. Medical consumption has been conceptualized in prior research from multiple perspectives. In health systems research, it is often defined in terms of the consumption of medical goods such as drugs, tests, and treatments, which are closely intertwined with healthcare service delivery and influence both patient costs and provider behavior [7]. More recent research has expanded this concept by adopting a consumer-oriented perspective that defines medical consumption as a process encompassing the acquisition, use, and disposal of medicines, while considering social, economic, and environmental dimensions [8]. This perspective emphasizes that individuals are not passive recipients of care but active agents who make decisions under constraints, including financial burden, access limitations, and system complexity.

Although individuals who are visually impaired constitute a significant proportion of the disabled population, their lived experiences and systemic barriers have received comparatively limited scholarly attention relative to other disability groups [2,9]. This gap is particularly important, given that visually impaired individuals often encounter compounded challenges related to medical expenses, insurance navigation, and healthcare accessibility, which directly influence health behaviors and outcomes [10,11].

The existing research on visual impairment has primarily focused on three domains. First, studies on assistive and smart technologies have examined GPS navigation systems, digital interface design, and AI-driven accessibility innovations to enhance mobility and autonomy [12,13]. Second, research on social participation has explored cultural and recreational inclusion, highlighting leisure activities as pathways for empowerment and well-being [14,15]. Third, educational and rehabilitation studies have emphasized lifelong learning, skill development, and capacity building among visually impaired adults [10,16,17]. Although these strands yield valuable insights, they tend to focus on either technological innovation or individual-level adaptation [18]. Consequently, limited attention has been given not only to how multilevel social, organizational, community, and policy environments collectively shape healthcare experiences, but also to how these processes are reflected in medical consumption, particularly in terms of how individuals perceive, navigate, and articulate healthcare-related costs, access, and system constraints.

From a health education perspective, this limitation is significant. Health behaviors are embedded within layered social and structural contexts, and interventions that target only individual knowledge or skills are unlikely to produce sustainable change without supportive institutional and policy environments. Therefore, disability scholars are increasingly calling for ecological approaches that connect personal adaptation processes with broader systemic and environmental determinants of health [1,15].

The Social Ecological Model (SEM) provides a theoretically robust and intervention-oriented framework for addressing this complexity by conceptualizing health-related behaviors as the product of interactions across multiple levels: individual, interpersonal, organizational, community, and policy/environmental [19,20,21]. Although SEM has been widely applied in public health and health promotion research, its use in disability-related healthcare discourse [22], particularly in digital environments, remains limited [23,24]. Moreover, little empirical research has examined how individuals with visual impairments articulate healthcare-related challenges across ecological levels in online spaces where lived experiences are often expressed. Furthermore, few studies have examined how medical consumption is discursively constructed across SEM levels in an online context.

In addition, while recent studies have begun to adopt computational approaches to analyze online health discourse, limited research has integrated large language model (LLM)-based classification with theory-driven frameworks such as SEM. In particular, the development of structured, theory-informed prompts to guide LLM interpretation remains underexplored despite its importance in improving interpretability and consistency in automated text analysis.

To address this gap, the present study used a text-mining method to examine online discourse about medical consumption among visually impaired individuals in the r/Blind community through the lens of SEM. Specifically, this study investigates how concerns related to medical expenses, insurance systems, and healthcare access are distributed across ecological levels and how these levels intersect within the digital discourse. By incorporating an LLM-based classification approach guided by a theory-driven prompt design, this study also demonstrates how abstract theoretical frameworks can be operationalized into interpretable and reproducible analytical procedures. This study advances health education scholarship in three ways by combining computational text analysis with ecological theory. First, it extends the ecological perspective to the discourse on digital disability. Second, it provides empirical evidence regarding structural and policy-level barriers that shape healthcare experiences. Third, it offers multi-level insights that can inform integrated health education strategies, institutional practices, and policy interventions to improve healthcare equity in visually impaired populations.

## 2. Methods

### 2.1. Data Source

This study analyzed user-generated content (UGC) from r/Blind, a Reddit subreddit dedicated to individuals who are blind or visually impaired. Reddit is a large online discussion platform composed of topic-specific communities known as subreddits, where users can share information and interact anonymously with others [25]. Most posts and comments are publicly accessible, allowing researchers to observe naturally occurring online discussions.

The r/Blind community primarily consists of individuals who are blind or visually impaired, with approximately 30,000 members. In addition to visually impaired users, the community includes family members, professionals, and individuals interested in issues related to visual impairment. According to the community descriptions:

*“Welcome to the hub for blind and visually impaired redditors. We are a support community for people who are blind or visually impaired, their friends and family, those who work with the blind, and those who are just curious.”*

As this description reflects, r/Blind is a supportive online space for sharing diverse experiences, knowledge, and information related to visual impairment while fostering mutual understanding and community engagement among its members.

### 2.2. Data Collection and Filtering

The data were collected in July 2025 using Python (3.13.5) (via Reddit’s official Application Programming Interface (API). This study focused on online discussions on medical consumption, particularly those related to medical expenses and health insurance.

To identify the relevant discussions, a keyword list related to medical expenses and insurance was developed. Two researchers with expertise in social media research generated a preliminary keyword list, which was subsequently refined through consultation with a medical professional specializing in visual impairment.

Keywords related to medical expenses included “medical expense,” “surgery cost,” “emergency room bill,” “medication cost,” “medical billing,” and “medical debt.” Health insurance-related keywords included “health insurance,” “insurance claim,” “insurance reimbursement,” “insurance copay,” and “high deductible plan.”

Because Reddit posts are typically unstructured and narrative, keyword searches may retrieve posts that are only partially related to a research topic. To improve the dataset’s topical relevance, an LLM was used to filter the collected posts. Specifically, OpenAI’s GPT-4.1-nano model was employed to evaluate whether each post was related to medical expenses or insurance issues. The following prompt was used:

*“Does the following Reddit post discuss medical expenses or insurance issues? Reply only with ‘Yes’ or ‘No’.”*

Posts classified as relevant by the LLM were retained for further analysis. To ensure reliability, two researchers independently reviewed the filtered results and confirmed their relevance to medical consumption.

Through this process, 79 original posts published before July 2024 were identified as relevant to medical consumption. Additionally, 349 associated comments were collected, resulting in a final dataset of 428 text entries. Each entry included the post URL, publication date, content type (post or comment), and full text of the discussion. Only English-language texts were included in the final dataset.

This study used publicly available Reddit data and did not involve any interactions with users or access to private information. All data were collected from openly accessible online discussions wherein users voluntarily shared content. To protect user privacy, no attempts were made to identify individuals, and all excerpts were presented in a de-identified manner, without including usernames or any personally identifiable information. In addition, excerpts were carefully selected and, where necessary, minimally paraphrased to reduce the risk of traceability while preserving their original meaning.

### 2.3. LLM-Based Text Classification and Application of the SEM

To examine how discussions of medical consumption are structured across different social contexts, this study applied the SEM as an analytical framework. The SEM conceptualizes health-related experiences as occurring across five interconnected levels: individual, interpersonal, organizational, community, and policy/environmental [26]. This framework provides a systematic basis for interpreting disability-related discourse within a multilayered social structure.

An LLM-assisted text classification approach was employed to classify the collected texts at the SEM level. LLMs can capture contextual meanings and semantic relationships in text, making them well-suited for categorizing complex social discourse beyond simple keyword-based approaches.

In the initial stage, a strictly theory-driven prompt was developed to ensure that the classifications closely adhered to the conceptual definitions of the SEM framework established by McLeroy, Bibeau [26]. This prompt emphasized explicit mention and context validation rules to minimize subjective interpretation and enhance interrater reliability. The prompt was implemented using the OpenAI GPT-5.3 model via the Python API with deterministic settings (temperature = 0) to ensure consistent and reproducible classification outputs. For comparative validation, an additional classification was conducted using the Google Gemini 1.5 Flash model (Web Paid Tier) with the same prompt structure. The model was used with deterministic settings (temperature = 0) to ensure consistent and reproducible classification outputs.

Despite the theoretically grounded design, initial classification results revealed discrepancies between the two LLMs, particularly in cases involving ambiguous or weak contextual cues. The inter-model agreement at this stage was relatively low (Cohen’s κ = 0.303–0.378 range across models), indicating inconsistencies in how each model interpreted the SEM framework under strict theoretical constraints.

To improve classification accuracy and consistency, the prompt was iteratively refined by incorporating stricter contextual constraints, including explicit keyword validation, exclusion rules for ambiguous expressions, and a hierarchical tie-breaking mechanism aligned with the SEM levels. The final prompt structure is presented in Table 1.

Following refinement, the classification process was repeated using both LLMs. The revised prompt improved alignment between model outputs and validated labels, with GPT-5.3 demonstrating higher performance (Accuracy = 56.8%, Cohen’s κ = 0.378) than Gemini 1.5 Flash (Accuracy = 43.2%, Cohen’s κ = 0.303).

Across the full dataset (N = 419), the two models agreed in 382 cases (91.2%) and disagreed in 37 cases (8.8%). All disagreements were manually adjudicated to ensure reliability. Two researchers independently reviewed each case according to the predefined SEM criteria and subsequently reached a consensus through discussion. This process assigned a final label to each disputed instance, producing a fully validated dataset.

## 3. Results

### 3.1. Distribution of Posts and Comments Across SEM Levels

**Figure 1:**
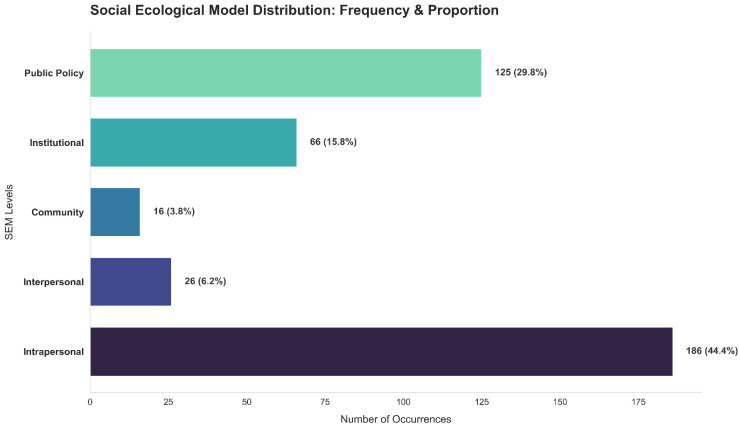
Distribution of Reddit documents across Social Ecological Model (SEM) levels.

The online discourse on medical consumption was categorized into the five levels of the SEM (Figure 1). Among the 419 text entries, the intrapersonal level accounted for the largest proportion (186 cases, 44.4%). This was followed by the public policy level with 125 cases (29.8%), the institutional level with 66 cases (15.8%), the interpersonal level with 26 cases (6.2%), and the community level with the lowest frequency (comprising 16 cases (3.8%)).

These findings indicate that discussions related to medical consumption among individuals who are blind or visually impaired are primarily centered on personal experiences and internal factors such as individual perceptions, coping strategies, and financial concerns. Simultaneously, a substantial portion of the discourse also reflects structural dimensions, particularly issues related to public policy, including health insurance systems and eligibility constraints.

In contrast, discussions related to community-level contexts (such as environmental accessibility or local infrastructure) and interpersonal interactions (such as support from family or caregivers) appear relatively limited. The relatively low frequency of community- and interpersonal-level discourse suggests that online discussions are less oriented toward relational or geographically bounded contexts.

### 3.2. Keyword Analysis by Levels of the SEM

To better understand the meanings embedded in the medical consumption discourse as classified through SEM, a keyword analysis was conducted for documents categorized at each ecological level. Using the term frequency–inverse document frequency (TF-IDF) approach, the top 20 salient keywords were extracted for each level (Table 2). By identifying the most significant words across the SEM levels, this analysis aimed to provide a structured understanding of how visually impaired individuals discuss their medical consumption.

**Figure 2:**
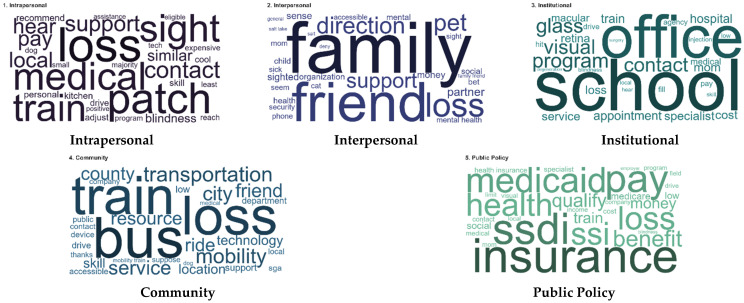
Wordclouds of distinctive keywords across five SEM levels.

Additionally, word clouds were generated from the TF-IDF results to visually represent the most prominent keywords at each SEM level (Figure 2). At the intrapersonal level, keywords such as *blindness*, *assistive*, *medical*, *patch*, and *retinal* highlight a strong focus on individual health conditions, treatment experiences, and personal coping strategies. In contrast, the interpersonal level is characterized by terms such as *family*, *friend*, *partner*, and *support*, reflecting the role of close social relationships in shaping medical experiences.

At the institutional level, frequently occurring keywords, including hospital-related services (e.g., office, specialist, and program) and condition-specific terms (such as *glaucoma* and *macular*) suggest an emphasis on formal healthcare systems and service delivery processes. Community-level features included keywords such as *buses*, *transportation*, *location*, and *mobility*, indicating the importance of environmental accessibility and transportation infrastructure for accessing medical services.

Finally, the public policy level includes terms such as *insurance*, *medicaid*, *ssdi*, *qualify*, and *benefit*, which reflect structural and systemic factors, particularly those related to health insurance systems and financial eligibility.

### 3.3. Core Issues and Implications of Medical Discourse Across Social Ecological Levels

Building on the preceding analyses of keyword patterns and distributions, this section presents an in-depth qualitative interpretation of representative excerpts from posts and comments classified at each level of the SEM. Through a close reading of these texts, this study sought to identify how experiences of medical access and healthcare consumption among visually impaired people are articulated and contextualized in online discourse. Table 3 summarizes the key themes for each SEM level, integrating the most salient keywords and representative excerpts.

At the intrapersonal level, discussions primarily revolved around psychological anxiety associated with vision loss, emotional expressions of identity reconstruction, and the process of coping with everyday constraints. For instance, the statement *“I’m scared to lose what’s left of my sight, but I’m trying to stay positive”* illustrates the coexistence of fear and resilience in facing progressive vision loss. Another user wrote, *“I’m choosing not to own pets. As much as I love animals, I just don’t think I could handle the responsibility of owning one*”, thus highlighting the inner conflicts between autonomy, responsibility, and daily functional limitations. These narratives reflect how individuals negotiate personal agency and self-identity within the boundaries of visual and economic realities.

At the interpersonal level, the presence or absence of family and social support emerged as a critical theme. The post *“My family rejected me after I lost my vision”* conveys the emotional rupture and social isolation that can accompany the onset of blindness, while another comment (*“We are your family now. DM me anytime”*) demonstrates how online communities can function as surrogate social support systems, providing emotional solidarity and non-physical forms of companionship. Such exchanges underscore the dual nature of interpersonal discourse, encompassing both the relational loss and the compensatory functions of virtual support networks.

At the institutional level, discourse is centered on the accessibility and effectiveness of formal service systems, such as rehabilitation centers and structured training programs. Certain references highlight the perceived reliability and necessity of structured institutional programs (e.g., “*Louisiana Center for the Blind, Colorado Center for the Blind, and Blind Incorporated are training centers that are well recognized for providing intensive rehabilitation training*”). Similarly, statements like “*Learning how to be blind, like going to a blind training center or receiving structured guidance from trained professionals, may be useful*” emphasized the importance of individualized rehabilitation and skill development, while demonstrating that online communities also serve as information hubs for navigating service systems and available resources.

At the community level, spatial accessibility and geographic inequality in available resources were identified as central issues [27]. For example, *“No public transport here. I feel trapped in my neighborhood”* reflects both physical and psychological isolation stemming from inadequate public transportation options. In contrast, another post (*“Our local Lions Club purchases items like these for individuals who need them in the community”*) illustrates how informal community-based organizations can act as supplemental resource providers, bridging local gaps in formal service provision.

At the public policy level, administrative complexity and restrictive eligibility criteria in welfare programs such as Medicaid and SSI/SSDI were frequently discussed. Statements like “*I was denied SSI because I have a roommate”* reveal the rigidity of current welfare policies that fail to account for the actual living conditions of recipients. Another comment (“*I am on a government-supported employment program (federal work study) and have applied to multiple jobs but have not received any interviews*”) reflects frustration with the structural barriers to employment and the resulting limitations on economic independence. Collectively, these excerpts emphasize the need for multidimensional, integrative approaches to healthcare policy and service design that reflect the lived realities of visually impaired individuals within intersecting personal, social, and structural contexts.

### 3.4. Keyword Similarity Analysis Across Levels of the SEM

**Figure 3:**
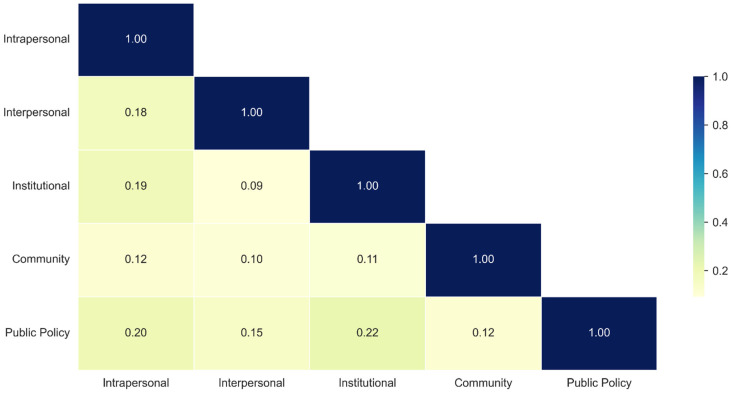
Jaccard similarity of TF-IDF keywords across SEM levels.

Based on the TF-IDF keyword extraction results, the similarity of keyword sets across the five levels of SEM was quantitatively examined using the Jaccard similarity coefficient (Figure 3). The Jaccard similarity measures the degree of overlap between two sets, ranging from 0 to 1, where higher values indicate greater similarity in keyword composition. This analysis aimed to assess thematic interconnectedness and distinctiveness among SEM levels within the medical consumption discourse, thereby providing empirical evidence to inform future institutional designs and community-based intervention strategies.

The analysis revealed that public policy and institutional levels exhibited the highest similarity, with a Jaccard coefficient of 0.22. This result indicates that both levels predominantly address structural issues such as public welfare systems, service accessibility, and medical or social resources. Overlapping keywords, such as insurance, Medicaid, program, and resources, suggest a strong thematic linkage between policy frameworks and the institutions responsible for their implementation, highlighting the intertwined nature of welfare system design and organizational service delivery.

In contrast, the intrapersonal and interpersonal levels showed a moderate degree of similarity (0.18), reflecting the way personal, psychological, and physical experiences are often articulated through interactions with family members or close acquaintances. Keywords, such as *loss*, *support*, *family*, and *mom*, indicate that individual experiences of vision loss or medical challenges are frequently embedded in the emotional and relational contexts of care and dependency.

However, the institutional and interpersonal levels exhibited the lowest similarity (0.09), representing the lowest overlap among all level pairs. This finding suggests that discussions on formal service systems are relatively distinct from those on interpersonal interactions and relationship-based experiences. Similarly, the community level showed consistently low similarity with other levels (ranging from 0.10 to 0.12), implying that community-related discourse forms a relatively independent thematic structure.

## 4. Discussion

This study examines how visually impaired individuals discuss healthcare-related experiences in online communities by applying SEM to user-generated content from the r/Blind subreddit. By analyzing discussions across multiple ecological levels, the findings offer insights into how healthcare challenges are perceived and expressed in digital environments.

One key finding was the prominence of intrapersonal discourse. A substantial portion of the discussion is focused on individual experiences, including emotional responses, coping strategies, and financial concerns related to vision loss and medical care. This pattern suggests that visually impaired individuals often articulate their healthcare experiences through personal narratives that combine psychological adaptations and practical challenges. For example, users frequently mentioned having anxiety related to vision deterioration as well as concerns related to affordability and day-to-day management of health conditions. These findings are consistent with prior research, indicating that blindness involves complex psychological and social adjustment processes influenced by multiple contextual factors [28].

At the same time, a notable portion of the discourse also reflects structural dimensions, particularly at the public policy and institutional levels. The discussions in these categories included references to health insurance systems, eligibility criteria, and access to formal services. Rather than suggesting that online discourse is primarily shaped by structural issues, these results indicate that structural concerns represent an important component in how individuals interpret and contextualize their healthcare experiences. In this sense, personal and structural dimensions appear to coexist within the same narrative, rather than operating as separate domains.

In contrast, discussions at the interpersonal and community levels are relatively limited. This pattern may be related to the characteristics of online platforms, such as Reddit, where interactions are often anonymous and organized around shared interests rather than geographically bounded relationships. As a result, the discourse may be less oriented toward local community contexts or sustained interpersonal relationships. However, because this interpretation is based on frequency patterns, it should be approached with caution.

### 4.1. Theoretical Contributions

This study contributes to the literature on disabilities and health discourse in several ways. First, it expands the use of SEM to analyze digital discourse among visually impaired individuals. While earlier studies have used ecological perspectives to examine participation and well-being among visually impaired populations [29], most have relied on interviews or surveys. By analyzing user-generated online discussions, this study demonstrates that SEM-based ecological layers can also be identified in naturally occurring digital communication.

Second, this study contributes methodologically by integrating the LLM-based classification with a theory-driven prompt design. Rather than treating LLMs as black box tools, this study developed a structured prompt based on SEM definitions, including explicit inclusion criteria, exclusion rules, and contextual validation guidelines. This approach illustrates how theoretical frameworks can be operationalized into interpretable prompt structures, enabling a more consistent and transparent classification of complex social discourse.

Finally, the findings provide empirical insights into how healthcare-related experiences are expressed by visually impaired individuals in online environments. The results suggest that these experiences are primarily articulated through intrapersonal narratives, while also incorporating structural considerations such as policy and institutional constraints. This highlights the coexistence of personal and structural dimensions in the digital health discourse, offering a more nuanced understanding of how healthcare challenges are interpreted online.

### 4.2. Practical Implications

The findings of this study have several practical implications for policymakers, healthcare providers, and social service organizations. First, the emphasis on policy and institutional discourse underscores the need for more accessible and transparent healthcare systems for visually impaired individuals. Simplifying administrative procedures, improving information access, and providing clearer guidance on healthcare benefits may help reduce the structural barriers often discussed in online communities. Second, the findings indicate that online communities can serve as valuable platforms for information exchange and peer support among the visually impaired. Policymakers and healthcare organizations should consider using online platforms to share health-related information, offer guidance for medical services, and facilitate community-based support networks. Third, the results suggest that healthcare interventions for the visually impaired individuals should adopt a multilevel approach. Programs focusing solely on individual rehabilitation or psychological support may overlook broader institutional challenges. Effective healthcare policies should thus combine personal support services with institutional reforms that improve accessibility and affordability and reduce administrative complexity.

### 4.3. Limitations and Future Research

Despite its contributions, this study has several limitations. First, the dataset was collected from a single online community, which may limit the generalizability of the findings. Although r/Blind is one of the largest online communities for visually impaired individuals, the experiences shared there may not fully reflect the perspectives of all visually impaired populations, particularly those who do not actively participate in online communities.

Second, the study focused exclusively on English-language posts, potentially excluding discussions from non-English-speaking users. Future research could expand this scope by analyzing multilingual datasets or examining online communities across different cultural contexts.

Third, this study employed a cross-sectional design, collecting data at a single point in time. Therefore, it does not capture temporal changes in discourse or shifts in how healthcare-related experiences are discussed. Future research could adopt longitudinal designs to examine how online discourse evolves over time, or in response to policy changes.

Fourth, although the classification of discourse using the SEM was supported by LLM-assisted categorization and human validation, some interpretive limitations remain inherent in automated text classification. Future studies could further strengthen reliability by incorporating larger-scale human coding, cross-platform validation, or hybrid approaches that combine multiple analytical methods, such as topic modeling, semantic embedding, or network analysis.

Finally, future research could extend this study by comparing discourses across multiple online communities, examining differences between countries, or analyzing variations across platforms with different user characteristics. Such approaches would provide a more comprehensive understanding of how healthcare-related experiences of visually impaired individuals are expressed in diverse digital contexts.

## 5. Conclusions

This study examined how healthcare-related experiences are articulated online among visually impaired individuals by applying SEM to Reddit user-generated content. The findings demonstrate that the medical consumption discourse is predominantly expressed at the intrapersonal level, reflecting personal coping processes, emotional responses, and financial concerns, while also incorporating structural dimensions related to public policy and institutional systems. These results highlight that individual and structural perspectives are not mutually exclusive but coexist within the same narratives, offering a more nuanced understanding of how healthcare challenges are interpreted in digital environments.

In addition to empirical findings, this study contributes methodologically by demonstrating the application of a theory-driven LLM-based classification approach. By developing a structured prompt grounded in SEM definitions and incorporating explicit validation rules, this study demonstrated how abstract theoretical frameworks can be translated into reproducible and interpretable computational procedures. This approach provides a foundation for future research that integrates LLMs with theory-based analysis in health discourse and underscores the potential of combining human-centered theory with scalable text analysis methods in health education and disability research.

## Figures and Tables

**Table 1:** Prompt Structure for Social Ecological Model (SEM)-Based Text Classification with Explicit Context Validation Rules.

SEM Level	Inclusion Criteria	Exclusion Rules	Key Examples
Public Policy	Explicit reference to government programs, insurance systems, or legal policies	Private/ambiguous insurance, general “benefits.”	Medicaid, Medicare, eligibility
Institutional	Interaction with formal organizations or service delivery systems	Past experiences without a service context	Hospital, appointment, provider
Community	Physical or environmental context affecting access	Words used in unrelated forms (e.g., “training”)	Transportation, neighborhood
Interpersonal	Direct interaction with identifiable individuals	Generic or abstract social terms	Family, caregiver
Intrapersonal	Individual-level emotions, perceptions, or abilities	—	Stress, coping, knowledge

**Table 2:** Top 20 Term frequency–inverse Document Frequency Keywords by Social Ecological Model Level.

Intrapersonal	Interpersonal	Institutional	Community	Public Policy
animal	age	blindness	bus	benefit
assistive	blindness	cost	city	glass
blindness	cat	drive	cost	health
center	direction	glass	county	health insurance
computer	dog	glaucoma	dog	income
contact	family	injection	drive	insurance
dog	friend	loss	expense	loss
drive	health	low	friend	low
hear	loss	macular	location	medicaid
loss	partner	medical	loss	medical
medical	pet	normal	mobility	money
patch	reach	office	money	pay
pay	real	pressure	raise	qualify
personal	sense	program	raise money	social
recommend	sight	school	ride	specialist
retinal	sight loss	service	service	ssdi
sight	social	specialist	support	ssi
skill	software	surgery	technology	surgery
support	stick	train	train	train
train	support	visual	transportation	visual

**Table 3:** Summary of Key Issues and Exemplary Quotes across SEM Levels.

SEM Level	Key Exemplary Excerpts	Main Issues
Intrapersonal	I’m scared to lose what’s left of my sight, but I’m trying to stay positive. I’m choosing not to own pets because I don’t think I could handle the responsibility.	Emotional adaptation, personal coping, and self-regulation
Interpersonal	My family rejected me after I lost my vision. We are your family now. DM me anytime.	Family rejection and peer-based emotional support
Institutional	Louisiana Center for the Blind and similar training centers provide structured rehabilitation programs. Learning through formal training centers or professional instruction may be helpful.	Access to structured training services and institutional support
Community	No public transport here. I feel trapped in my neighborhood. Our local Lions Club provides assistive resources for people in the community.	Transportation barriers and community-level resource availability
Public Policy	I was denied SSI because I have a roommate. I am on federal work study but have not received any job opportunities.	Barriers related to eligibility and access to government support programs

## Data Availability

The data used in this study are not publicly available due to restrictions related to data management and privacy considerations.
